# When should a patient with prior COVID-19 infection be placed in isolation precautions if readmitted months later?

**DOI:** 10.1017/ice.2020.408

**Published:** 2020-08-06

**Authors:** Leonard A. Mermel

**Affiliations:** 1 Department of Medicine, Warren Alpert Medical School of Brown University; 2 Division of Infectious Diseases, Rhode Island Hospital; 3 Department of Epidemiology and Infection Control, Rhode Island Hospital

*To the Editor—*In our hospital system, we do not consider the possibility of reinfection unless a patient had a positive SARS-CoV-2 PCR detected in a nasopharyngeal or nasal swab specimen >3 months from the first such positive PCR test or before then if immunocompromised.^1^ However, we have had patients previously admitted with COVID-19 pneumonia, discharged with waxing and waning SARS-CoV-2 PCR positivity over the following 12–13 weeks, and found to be PCR positive with high PCR cycle target thresholds when they were readmitted. These patients’ initial, severe infection has led to prolonged PCR positivity.^2^

Extracellular vesicles released from infected cells may promote infection prior to the development of an immune response.^3^ However, it is possible that RNA and/or other viral fragments released from cells over the days and weeks thereafter continue to prime the immune system^4–6^ such that reinfection during this time is unlikely, as it may be for weeks thereafter. If true, it makes more sense to not consider reinfection in immunocompetent patients until >3 months after the last positive SARS-CoV-2 PCR result rather than the first one. Such a policy would have profound implications: it would reduce the need for personal protective equipment that otherwise would be used for placing such patients in isolation precautions if readmitted. Future research is needed to determine whether viral fragments released from cells after active infection has resolved promote prolonged immunity to reinfection such that isolation precautions do not need to be reinstituted until 3 months after the last positive SARS-CoV-2 test.
